# Integration of Kinetic Analysis of Reaction Curve with a Proper Classical Approach for Enzymatic Analysis

**DOI:** 10.1100/2012/969767

**Published:** 2012-05-03

**Authors:** Xiaolan Yang, Gaobo Long, Hairong Jiang, Pu Liao, Fei Liao

**Affiliations:** ^1^Unit for Analytical Probe and Protein Biotechnology, College of Laboratory Medicine, Chongqing Medical University, Chongqing 400016, China; ^2^College of Pharmacy and Bioengineering, Chongqing University of Science and Technology, Chongqing 400054, China; ^3^Department of Clinical Laboratory, The Third People's Hospital of Chongqing, Chongqing 400014, China

## Abstract

For enzymatic analysis to quantify a substrate or enzyme, kinetic analysis of reaction curve can be integrated with a proper classical approach. For their integration, they should have consistent slopes and intercepts of linear response and an overlapped region of analyte quantities measurable under optimized conditions. To quantify a substrate after optimizations of tool enzyme activity and reaction duration, the equilibrium method works when the reaction is completed within the reaction duration; otherwise, kinetic analysis of reaction curve applies providing at least seven data with sufficient consumption of substrate. To quantify an enzyme after optimizations of initial substrate concentration and reaction duration, the classical initial rate method works when an estimated initial rate locates within the linear range; otherwise, kinetic analysis of reaction curve applies after the conversion of the quantification index with optimized parameters. This integration strategy has ideal linear ranges and practical efficiency for quantifying an enzyme at moderate substrate levels and for quantifying a substrate at moderate cost on tool enzyme; it has promise to simultaneous assays of multiple enzymes in one reaction vessel each time and ,thus, potential applications to concurrently quantify multiple serum enzymes, screen inhibitors against multiple enzyme targets, and detect multiple serum components by enzymeimmunoassay.

## 1. Introduction

In biomedical laboratories, enzymatic analysis is routinely practiced to quantify enzyme or substrate [1–4]. For quantifying an enzyme, the classical initial rate method is commonly utilized with reaction data of small consumption percentage of substrate; it has favorable lower limit of linear response, but always suffers low upper limit of linear response [3, 4]. For quantifying a substrate, the equilibrium method and the classical kinetic method are practical approaches [3–6]. The equilibrium method in common use determines the difference between the initial signal before enzyme reaction and the final signal after the completion of enzyme reaction; it has a desirable lower limit of linear response and reasonable resistance to variations of tool enzyme activity but tolerates low analysis efficiency. The classical kinetic method makes use of the response of initial rates to substrate concentrations; it has ideal analysis efficiency but suffers narrow linear range and high sensitivity to variations of tool enzyme activity. Therefore, for enzymatic analysis, these classical approaches are still beyond satisfaction.

A practical alternative approach for quantifying an enzyme or substrate is kinetic analysis of reaction curve *via* one of the following two ways [7–22]. The first employs an integrated rate equation with the predictor variable of reaction time for least square fitting to a reaction curve after data transformation [11–22], the second adopts numerical integration of differential rate equation(s) to calculate reaction curves for least square fitting to reaction curve under analysis [9, 23, 24]. For quantifying enzyme by kinetic analysis of reaction curve, initial rate or maximal reaction rate (*V*_*m*_) can be estimated with Michaelis-Menten constant (*K*_*m*_) fixed after optimization. For quantifying a substrate by kinetic analysis of reaction curve, the final signal after the completion of enzyme reaction is predicted with reaction data far before the completion of reaction, while the initial signal before enzyme reaction is still determined by experimentation. This alternative approach for quantifying an enzyme or substrate has an upper limit of linear response much higher than that by classical approaches besides favorable cost.

For kinetic analysis of reaction curve to quantify an enzyme or substrate, however, there should be at least seven data of steady-state reaction with changes of detected signals over three times of random errors and should be over 35% decrease in the instantaneous rate at the last steady-state datum under analysis or large consumption of substrate [7, 20, 22, 25, 26]. For practical efficiency, thus, kinetic analysis of reaction curve for quantifying an enzyme or substrate mainly tolerates unfavorable lower limit of linear response [12, 13, 19, 21]. Kinetic analysis of reaction curve requires lower substrate consumption percentage for quantifying a substrate than that by the equilibrium method [24, 25, 27–31] but applies to reaction data of higher substrate consumption percentage for quantifying an enzyme than that by the classical initial rate method. For enzymatic analysis, hence, kinetic analysis of reaction curve may be integrated with a proper classical approach, which throughout the context is the equilibrium method for quantifying substrate but the classical initial rate method for quantifying enzyme, and thus reaction data with consumption percentages of substrate over wider ranges can be processed. Such an integration strategy for enzymatic analysis, may concurrently have ideal linear range, desirable efficiency and favorable cost on substrate or tool enzyme. 

Before we reported an integration strategy in 2009 to quantify enzyme with wonderful performance [25], the quantification of an enzyme always employs just one approach; any integration strategy for quantifying a substrate has not been reported yet up to date. In general, classical approaches for enzymatic analysis already fulfill the works in biomedical laboratories but there will be the following challenges on enzymatic analysis in the future. (a) To further enhance efficiency to measure enzyme activities during the quantification of serum enzymes and the screening of inhibitors against multiple enzyme targets, simultaneous assay of multiple enzymes in one reaction solution is an absorbing option. Suitable chromogenic substrates at moderate levels should be used in one reaction solution for negligible inhibition actions of chromogenic substrates and their contaminants; how to expand linear ranges for enzyme activity assays at moderate substrate levels becomes a challenge. (b) Enzyme linked immunosorbent assay (ELISA) is widely practiced in biomedical laboratories, but tolerates low analysis efficiency and narrow quantifiable range. To accelerate ELISA, simultaneous assays of two components with two enzyme labels in one reaction vessel have long been proposed, but no reported methods are practical [32–35]. How to expand quantifiable ranges for ELISA with chromogenic substrates at moderate levels is a challenge no matter single or two components are quantified. Certainly, the integration strategy may be a solution to such challenges on enzymatic analysis.

Chemometrics for the integration strategy of enzymatic analysis is sophisticated even it does not require multiple variable calibration or complicated pattern recognition. Herein, based on our researches, we reviewed and discussed chemometrics for the integration strategy to quantify an enzyme or substrate in a general manner.

## 2. Prerequisites of the Integration Strategy

For the integration strategy to quantify an enzyme or substrate as the analyte in different samples, the same signals under the same analysis conditions should be continuously recorded. Moreover, the integration strategy is applicable only to a sample whose analyte quantity is over the lower limit of linear response by the selected classical approach but below the upper limit of linear response by kinetic analysis of reaction curve. In general, the initial signal before enzyme reaction, the first recorded datum after preset lag time, the last recorded datum after preset reaction duration, and instantaneous reaction rates can be collected to roughly approximate analyte quantity in a sample for judging the applicability of the integration strategy.

For the integration strategy to a sample, a proper classical approach is applied when the analyte quantity locates in the linear rang of the classical approach; otherwise, kinetic analysis of reaction curve is utilized when the analyte quantity locates in its linear rang [24, 25, 27–30]. For enzymatic analysis, therefore, the integration strategy is predominantly characterized by selecting different approaches for processing reaction data with different samples. In practice, however, analyte quantity in a sample is unknown before data are processed with a proper approach. To quantify an analyte by the integration strategy, an iterative selection of a proper approach for processing enzyme reaction data is thus mandatory but laborious. However, as we discussed below, a switch threshold can be developed to select a proper approach for processing reaction data based on simple qualitative comparison. Therefore, the integration strategy for enzymatic analysis in routine practice can be established providing the following prerequisites are simultaneously satisfied [25, 27–30].

1. Kinetic analysis of reaction curve is validated for quantifying an enzyme or substrate and has a slope and intercept of linear response consistent with those by the classical approach, respectively.
2. There is an overlapped region of analyte quantities measurable reliably by both a proper classical approach and kinetic analysis of reaction curve for a continuous quantifiable range of analyte quantity.
3. A switch threshold is derived from changes of detected signals for an analyte quantity within the overlapped region to select the classical approach or kinetic analysis of reaction curve for processing reaction data based on simple qualitative comparison.

During enzyme reaction, there will be a continuous decrease in detected signals for the substrate while a continuous increase in detected signals for a stable product. After the first and second prerequisites are met, reaction data for the upper limit of linear response by a proper classical approach should be suitable for kinetic analysis of reaction curve. With preset reaction duration, thus, the change of detected signals for any analyte quantity within the overlapped region can serve as the switch threshold to select a proper approach for processing reaction data. Namely, the classical approach should be used when such a change with a sample is below the switch threshold, otherwise, kinetic analysis of reaction curve should be employed. Based on our experience, kinetic analysis of reactive curve yields results with larger uncertainty when analyte quantities are close to its lower limit of linear response. Therefore, with preset reaction duration, the change of detected signals for an analyte quantity that is close to or slightly lower than the upper limit of linear response by a proper classical approach is adopted as the switch threshold; in this case, approaches to the establishment of the integration strategy are discussed below. 

## 3. Approaches to the Integration Strategy

To meet the first prerequisite, parameters for kinetic analysis of reaction curve should be optimized and the quantification index by both approaches should have the same physical meaning. The quantification index by kinetic analysis of reaction curve is preferable to be converted into that by the classical approach when these two approaches have different quantification indexes. Therefore, in general, the first and second prerequisites can be met *via* optimizing the following factors: (a) parameters to validate kinetic analysis of reaction curve, (b) parameters to convert the quantification index of analyte quantity by kinetic analysis of reaction curve into that by a proper classical approach, so that slope and intercept of linear response can be consistent with those by a proper classical approach, respectively, and (c) conditions to produce an overlapped region of analyte quantities measurable by both approaches.

### 3.1. Approaches to the First Prerequisite

To quantify a substrate, the equilibrium method is robust but consumes a lot of time to determine the final signal after the completion of enzyme reaction. For quantifying a substrate by kinetic analysis of reaction curve, the final signal after the completion of enzyme reaction predicted with data far before the completion of enzyme reaction has exactly the same physical meaning as that with the equilibrium method [16–22]. To quantify a substrate, thus, the quantification index by kinetic analysis of reaction curve is exactly the same as that by the equilibrium method; the first prerequisite of the integration strategy can be met as long as kinetic analysis of reaction curve is validated to quantify the substrate. In general, the following criteria apply to optimized parameters for validating kinetic analysis of reaction curve to quantify a substrate or enzyme: (a) there is consistency of the predicted final signal by kinetic analysis of reaction curve with that by the equilibrium method; (b) there is resistance of final signal and other parameters to reasonable variations in data ranges for analysis.

**Figure 1:**
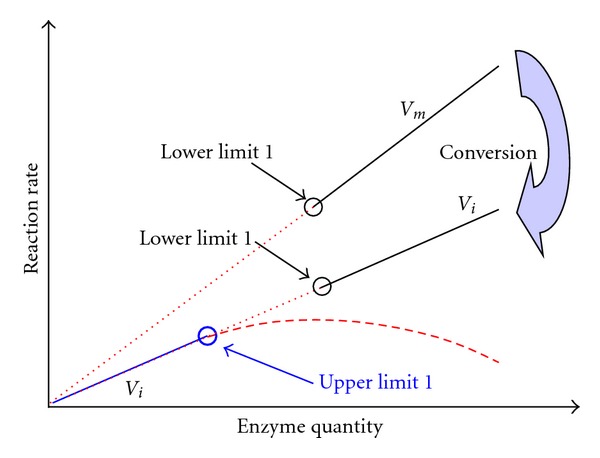
Conversion of *V*_*m*_ into initial rate (*V*_*i*_) to meet the second prerequisite for an integration strategy to quantify an enzyme.

To quantify an enzyme, the sole classical approach is the classical initial rate method. After kinetic analysis of reaction curve is validated for quantifying an enzyme with optimized parameters as described before [31], there are the following two situations to meet the first prerequisite due to the estimations of different quantification indexes by kinetic analysis of reaction curve. (a) The first situation is the estimation of *V*_*m*_ when either an integrated rate equation with the predictor variable of reaction time or numerical integration is used for kinetic analysis of reaction curve after data transformation. *V*_*m*_ can be converted into initial rate according to Michaelis-Menten rate equation with a preset substrate concentration (PSC) and other parameters after careful optimizations [25] (Figure 1). In the integration strategy, initial substrate concentration (*S*_0_) for quantifying *V*_*m*_ by kinetic analysis of reaction curve can be higher or lower than *K*_*m*_, but an optimized PSC is preferable to be about 93% of *S*_0_ to convert *V*_*m*_ into initial rate [24, 25, 27–30]. Such an optimized PSC is effective to meet the first prerequisite of the integration strategy for quantifying glutathione-S-transferase, butyrylcholinesterase, uricase, and gamma-glutamyltransferase [25, 27–30]. (b) The second situation is the estimation of initial rate itself. Usually, kinetic analysis of reaction curve can estimate initial rate *via* numerical integration of differential rate equation(s) to produce calculated reaction curves for least square fitting to reaction curve under analysis without data transformation. In this case, kinetic analysis of reaction curve has exactly the same quantification index as the classical approach, and there can be consistent slopes and intercepts of linear response between these two approaches to directly meet the first prerequisite for the integration strategy [24].

### 3.2. Approaches to the Second Prerequisite

After the first prerequisite is already met, the upper limit of linear response by the classical approach should be increased, or the lower limit of linear response by kinetic analysis of reaction curve should be decreased, for the required overlapped region of measurable analyte quantities. 

Thus, reaction duration and *S*_0_ for quantifying an enzyme while tool enzyme activity and reaction duration for quantifying a substrate become the principal factors for providing the overlapped region of measurable analyte quantities.

**Figure 2:**
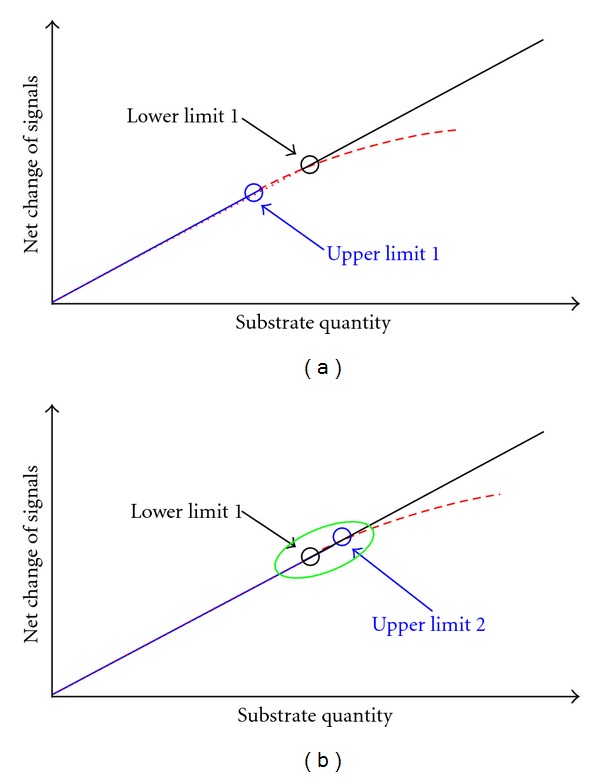
Optimization of activities of a tool enzyme to increase upper limit 1 to upper limit 2 by the equilibrium method for meeting the second prerequisite for an integration strategy to quantify a substrate.

For quantifying a substrate, shorter sampling intervals and/or lower tool enzyme activities can just slightly reduce the lower limit of linear response by kinetic analysis of reaction curve. For automated analysis in parallel and practical efficiency, sampling intervals should be about 10 s, while tool enzyme activity should enable the completion of reaction within reaction duration of about 5.0 min. To quantify a substrate by kinetic analysis of reaction curve, thus, the lower limit of linear response can hardly be reduced. In this case, the upper limit of linear response by the equilibrium method has to be increased *via* the increase in tool enzyme activity and/or reaction duration (Figure 2). 

**Figure 3:**
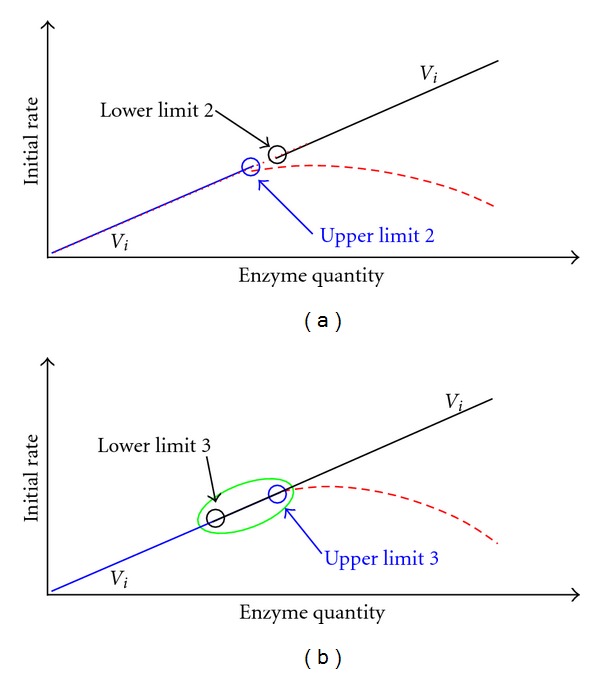
Optimizations of both *S*_0_ to increase the upper limit of the classical initial rate method and reaction duration to reduce the lower limit by kinetic analysis of reaction curve for meeting the second prerequisite for an integration strategy to quantify an enzyme (*V*_*i*_).

For quantifying an enzyme, the lower limit of linear response by kinetic analysis of reaction curve is usually inversely proportional to reaction duration. For favorable analysis efficiency, however, reaction duration should be just about 5.0 min. Thus, to meet the second prerequisite of the integration strategy for quantifying an enzyme, the upper limit of linear response by the classical initial rate method is desired to be increased by using higher *S*_0_. However, the use of *S*_0_ much higher than *K*_*m*_ usually requires longer reaction duration to realize the integration strategy for quantifying an enzyme [25]. With single enzyme reaction system, an optimized ratio of *S*_0_ to *K*_*m*_ is 0.5 through 2.5 for reaction duration of about 5.0 min as long as *K*_*m*_ of the enzyme of interest is not extremely high or low [25]. When the limitation by the quantification sensitivity, substrate solubility, and *K*_*m*_ and/or substrate inhibition requires the use of *S*_0_ beyond such a preferred range, reaction duration has to be prolonged to meet the second prerequisite (Figure 3). Minimum reaction duration for the integration strategy to quantify an enzyme can be approximated by theoretical derivation [25]. Results with uricase, butyrylcholinesterase, glutathione-S-transferase, and gamma-glutamyltransferase support the effectiveness of these optimization strategies [25, 28–31]. For enzyme-coupled reaction systems, experimental conditions with the classical initial rate method like tool enzyme activity and *S*_0_ can be used directly for the integration strategy, and sequential optimization of reaction duration usually can meet the second prerequisite [24].

Taken together, to meet the second prerequisite for the integration strategy, higher *S*_0_ can be used to quantify an enzyme while higher activities of tool enzyme can be used to quantify a substrate for favorable efficiency. When there are the limitations on *S*_0_ or tool enzyme activities, reaction duration has to be prolonged at the cost of efficiency.

## 4. Features of the Integration Strategy

For an analytical method, (a) its upper limit of linear response can be the highest level of an analyte to give quantification index with deviation from the linear plot below twice the standard error of estimateant (b) its lower limit of linear response can be the lowest level of an analyte to give quantification index with both deviation from the linear plot below twice the standard error of estimate and reasonable precision, that is, coefficient of variation (CV) below 20%. It is taken for granted that such definitions require consistent standard deviations along the whole response plot [26].

**Figure 4:**
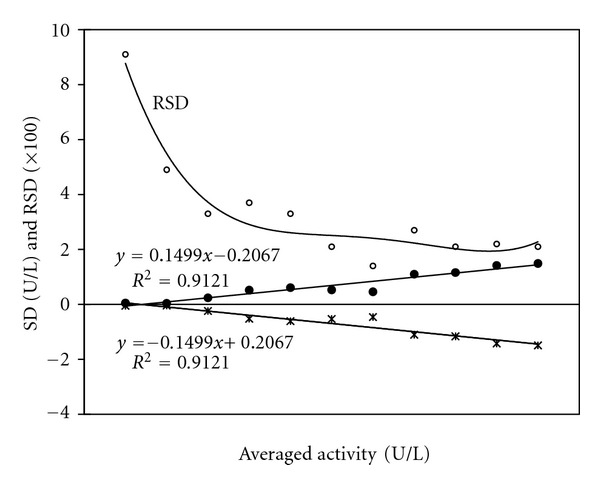
Association of standard deviations (SD) and relative SD (RSD) with averaged ALT activities by the integration strategy. Data were obtained by methods as reported before [24].

For the integration strategy, standard deviations are proportional to averages of analyte quantities and the overall standard error of estimate is always larger than that by the classical approach alone (Figure 4) [24, 25, 27–31]. Due to much wider linear range by the integration strategy, there must be sequential dilutions of a stock solution of the analyte to construct the linear response plot and thus inevitable larger standard error of estimate from the response plot. The integration strategy exactly employs the classical approach for analyte quantities at low levels. Therefore, the lower limit of linear response of the integration strategy is arbitrarily taken as twice the lower limit of linear response by the classical approach with CV below 10% if the standard error of estimate by the integration strategy is more than twice that by the classical approach; otherwise, it is still the lower limit of the classical approach with CV below 10% [24, 25, 27–29].

Our experimental results demonstrated that the integration strategy was effective to quantify enzymes like butyrylcholinesterase, glutathione-S-transferase, uricase, and gamma-glutamyltransferase [25, 28–31] and to quantify substrates including uric acid with uricase, reduced glutathione with glutathione-S-transferase, and ethanol with alcohol dehydrogenase [31]. In these representative applications, the integration strategy for enzymatic analysis concomitantly possesses (a) much wider linear ranges, (b) favorable analysis efficiency, (c) the use of practical substrate levels to quantify enzyme or desirable activities of tool enzymes to quantify substrate, and (d) resistance to common errors [24, 25, 27–31]. For quantifying an analyte, these four features can hardly be concurrently achieved by any proper approach alone. There is another integration strategy to quantify an enzyme by fitting a simplified integrated rate equation to reaction curve, but it requires *S*_0_ much lower than *K*_*m*_ to validate the integrated rate equation [11], which indicates that it can hardly be effective at *S*_0_ over enzyme *K*_*m*_.

To quantify multiple enzymes in each sample, to screen each inhibitor from a library against multiple enzyme targets and to quantify multiple components in each sample by ELISA, simultaneous measurement of multiple enzymes in one reaction solution is desired. For such enzymatic analyses in the future, moderate substrate levels have to been employed, and there should be more considerations on methodologies to quantify enzyme activities for expanded linear ranges at moderate substrate levels. We proposed an integration strategy for enzymatic analysis that possesses incomparable advantages to quantify single enzyme but still requires more verification. More importantly, this integration strategy has promise to simultaneous assays of multiple enzymes in one reaction solution with chromogenic substrates at moderate levels and may be fundamentals of enzymatic analysis methods of a new generation in the future.
